# Comparative Evaluation of a New Autonomous AI Agent Versus Frontier LLMs for AI-Generated Patient Information Sheets on Pediatric Pathologies

**DOI:** 10.3390/cancers18183047

**Published:** 2026-09-20

**Authors:** Zaid H. Khoury, Rata Rokhshad, Mohamed S. Sultan, Jeffery B. Price, Tiffany Tavares, Kimia Sadat Kazemi, Neda Najafimakhsoos, Ahmed S. Sultan

**Affiliations:** 1 Department of Oral Diagnostic Sciences & Research, School of Dentistry, Meharry Medical College, Nashville, TN 37208, USA; 2 Eli5a Technologies, 1206 Geneva, Switzerlandtavarest@uthscsa.edu (T.T.); 3 Department of Pediatric Dentistry, Loma Linda School of Dentistry, Loma Linda, CA 92350, USA; 4 Sparta Commodities, 1204 Geneva, Switzerland; 5 Division of Artificial Intelligence Research, University of Maryland School of Dentistry, Baltimore, MD 21201, USA; jbprice@umaryland.edu; 6 Department of Oncology and Diagnostic Sciences, University of Maryland School of Dentistry, Baltimore, MD 21201, USA; 7 Department of Comprehensive Dentistry, UT Health San Antonio School of Dentistry, San Antonio, TX 78229, USA; 8 College of Dentistry, University of Saskatchewan, Saskatoon, SK S7N 5E5, Canada; ckimia.kazemi@usask.ca; 9 Department of Biomedical and Neuromotor Sciences, Alma Mater Studiorum, University of Bologna, 40138 Bologna, Italy; neda1997najafi@gmail.com; 10 University of Maryland Marlene and Stewart Greenebaum Comprehensive Cancer Center, Baltimore, MD 21201, USA

**Keywords:** artificial intelligence, bot, autonomous, engagement, health information, large language models

## Abstract

Background: At present, there are very limited studies evaluating autonomous artificial intelligence (AI) agents in oral oncology or healthcare education, and no studies have directly compared traditional frontier large language models (LLMs) with autonomous AI agents in the field of pediatric oral oncologic pathology. This study evaluated the performance of AI LLMs and an autonomous AI scientific agent in generating engaging and accessible patient information sheets for common pediatric oral pathologic conditions and rare head and neck tumors. The development of high-quality patient education materials is particularly important in pediatric pathology because parents must often navigate complex and emotionally sensitive diagnoses, including rare tumors and developmental lesions, for which accessible, patient-friendly educational resources are frequently unavailable. AI-generated patient information sheets therefore represent a potential strategy to improve communication, understanding, and shared decision-making for families facing these uncommon conditions. Methods: AI-generated patient information sheets from popular frontier chatbots on various pediatric pathological conditions and rare tumors were evaluated by five platforms (Eli5a v2.0, ChatGPT-5.4, Claude Sonnet 4.6, Perplexity and Doximity), in a blinded fashion, by three expert evaluators using the Global Quality Score (GQS), DISCERN score, understandability score and actionability score using PEMAT, and the Flesch-Kincaid Grade Level. Because the same 20 conditions were assessed on every platform, observations were paired; platforms were compared using Friedman tests with paired Wilcoxon signed-rank post hoc tests and Holm correction, and interrater reliability was quantified using an absolute-agreement intraclass correlation coefficient (ICC). Results: Platform differences were significant for all five outcomes (all *p* < 0.001). Claude Sonnet 4.6 obtained the best overall information quality (GQS 4.25 ± 0.39), scoring significantly higher than all other platforms, while Perplexity, Eli5a v2.0 and ChatGPT-5.4 performed similarly to one another and better than Doximity. For information reliability, Doximity (DISCERN 70.50 ± 3.20) and Claude Sonnet 4.6 (70.25 ± 3.02) performed best, while Eli5a v2.0 recorded the lowest DISCERN score (54.50 ± 4.26). Eli5a v2.0 obtained the best results for patient-centered communication, with the highest understandability (PEMAT-U 95.05 ± 1.23) and readability (FKGL 5.45 ± 0.51) and an actionability score among the highest of the five platforms (PEMAT-A 89.00 ± 2.62, not significantly different from Perplexity or Doximity). Interrater absolute agreement for GQS was poor to moderate (ICC(2,1) = 0.236; ICC(2,3) = 0.481). Conclusion: No single platform was superior across all evaluated domains. Claude Sonnet 4.6 led in overall quality whereas Eli5a v2.0 achieved the highest understandability and the most appropriatereading grade level. Eli5a’s actionability was high but did not differ significantly from Perplexity or Doximity. These findings indicate a trade-off between information quality and reliability on one hand and lay accessibility on the other. Platform selection should therefore be matched to the communication task, and all AI-generated patient materials require clinician review before use.

## Simple Summary

This study compared five artificial intelligence frontier platforms on their ability to write patient information sheets about common and rare pediatric oral conditions. No single platform was best at everything. Claude Sonnet 4.6 produced the highest overall quality and, together with Doximity, the most reliable material. Eli5a v2.0, a newly developed autonomous AI agent, produced the clearest and most readable material, achieved an actionability score among the highest, and was the only platform to write at the reading level recommended for patient education. The results point to a trade-off between depth of information and ease of reading, suggesting that tool selection should follow the communication task rather than a single overall ranking.

## 1. Introduction

Large language models (LLMs) are generative artificial intelligence systems that process and generate language and have shown promise in a range of healthcare applications [1]. Natural language processing can support interactive tools that help users understand health information [2]. Some of these tools operate within ambient-intelligence environments that respond to users and context [3]. Since late 2022, general-purpose LLMs such as ChatGPT (OpenAI) have been explored as adjuncts for clinical practice and patient education, followed by multimodal systems capable of processing text, images, audio, and video. More recently, agentic AI systems have been developed to plan and execute multistep tasks with limited ongoing user input. Research on non-autonomous LLMs, including Claude (Anthropic), ChatGPT (OpenAI), Copilot (Microsoft), Gemini (Google), and Perplexity (Perplexity AI), has reported promising performance in several medical tasks. The public, particularly younger generations, increasingly uses LLMs and social media to obtain health information [4]. However, these platforms can also amplify misinformation and algorithmic bias, underscoring the need for careful evaluation and professional oversight [5,6].

Studies evaluating autonomous AI agents in oral oncology and healthcare education remain limited, and direct comparisons between frontier large language models (LLMs) and autonomous AI agents in pediatric oral oncologic pathology have not been performed [7]. This gap is particularly consequential because parents of children with rare oral and maxillofacial tumors often face complex, emotionally sensitive diagnoses for which accessible educational resources are lacking [8]. Autonomous AI agents are increasingly being developed to automate administrative and patient-facing healthcare tasks, with the potential to reduce clinician workload and physician burnout [9]. In head and neck oncology and pathology, where specialist shortages exist and patient-friendly educational resources for uncommon oral mucosal diseases and rare tumors are often unavailable, autonomous AI agents may serve as clinical adjuncts by generating educational materials and assisting with patient communication under appropriate human oversight [10,11]. A comprehensive narrative review characterized pediatric applications as a growing subset of the broader AI-in-dentistry literature but did not include patient information related to pediatric oral pathology [12]. Furthermore, molecular and genetic advances are introducing terminology such as microRNAs, non-coding RNAs, molecular signatures, and genetic alterations that may require careful explanation to patients and families [13,14]. The aim of this study was therefore to compare a newly developed autonomous AI agent with four frontier LLM platforms in generating patient information sheets for common pediatric oral pathologic conditions and rare head and neck tumors, with respect to information quality, reliability, readability, understandability, and actionability.

It was hypothesized that patient information sheets generated by an autonomous agent developed specifically for a lay audience would outperform those generated by general-purpose platforms across these five domains.

## 2. Methods

### 2.1. Overview of the Proposed Methodology

**Figure 1:**
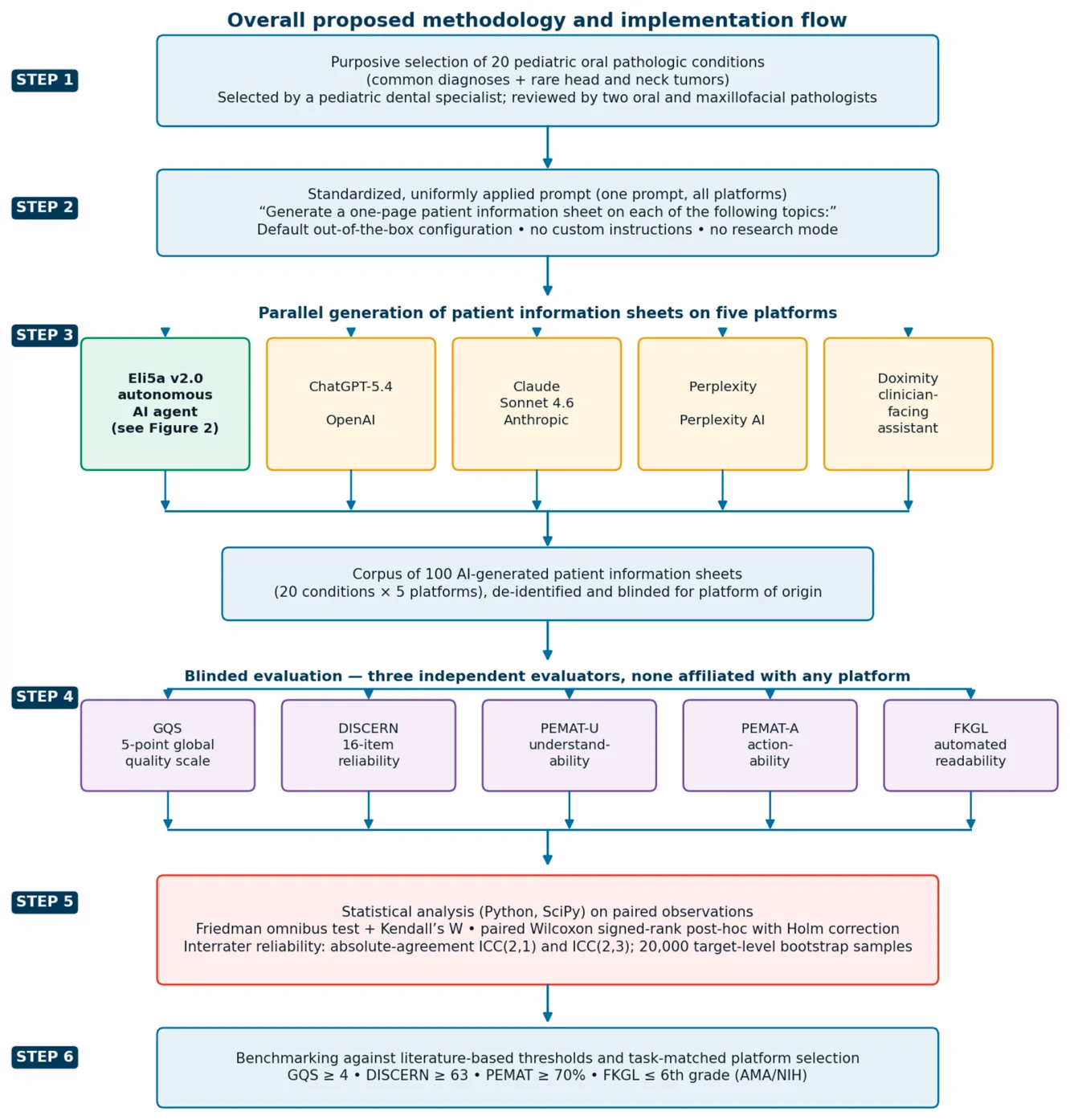
Overall proposed methodology and implementation flow of the comparative evaluation. Figure 1 description. Step 1, purposive selection of the 20 pediatric oral pathologic conditions, led by a pediatric dental specialist and reviewed by two oral and maxillofacial pathologists. Step 2, the single standardized prompt applied uniformly to all platforms under default configurations, without custom instructions, style customization, or research mode. Step 3, parallel generation of the patient information sheets by the five evaluated platforms, of which Eli5a v2.0 is the autonomous AI agent whose internal architecture is shown in Figure 2, yielding a corpus of 100 sheets that was de-identified and blinded for each platform. Step 4, blinded assessment of that corpus by three independent evaluators, none affiliated with any evaluated platform, using the GQS, DISCERN, PEMAT understandability, and PEMAT actionability instruments together with automated Flesch–Kincaid Grade Level scoring. Step 5, the statistical pipeline applied to the paired observations, comprising Friedman omnibus tests with Kendall’s W, paired Wilcoxon signed-rank post hoc tests with Holm correction, and absolute-agreement intraclass correlation coefficients with target-level bootstrap confidence intervals. Step 6, benchmarking of the outcomes against the literature-based thresholds used for task-matched platform selection.

The overall methodology of this study comprised six sequential stages, which are summarized in Figure 1. First, 20 pediatric oral pathologic conditions spanning common diagnoses and rare head and neck tumors were purposively selected. Second, a single standardized prompt was constructed and applied uniformly to every evaluated platform under default, out-of-the-box configurations. Third, patient information sheets were generated in parallel by all five platforms, namely the autonomous AI agent Eli5a v2.0 and the four frontier LLM platforms; ChatGPT-5.4, Claude Sonnet 4.6, Perplexity, and Doximity, producing a corpus of 100 sheets (20 conditions × 5 platforms). Fourth, the de-identified corpus was assessed in a blinded manner by three independent evaluators using the GQS, DISCERN, and PEMAT understandability and actionability instruments, together with automated Flesch–Kincaid Grade Level scoring. Fifth, the resulting paired observations were analyzed statistically. Sixth, the outcomes were benchmarked against literature-based thresholds in order to derive task-matched recommendations for platform selection.

**Figure 2:**
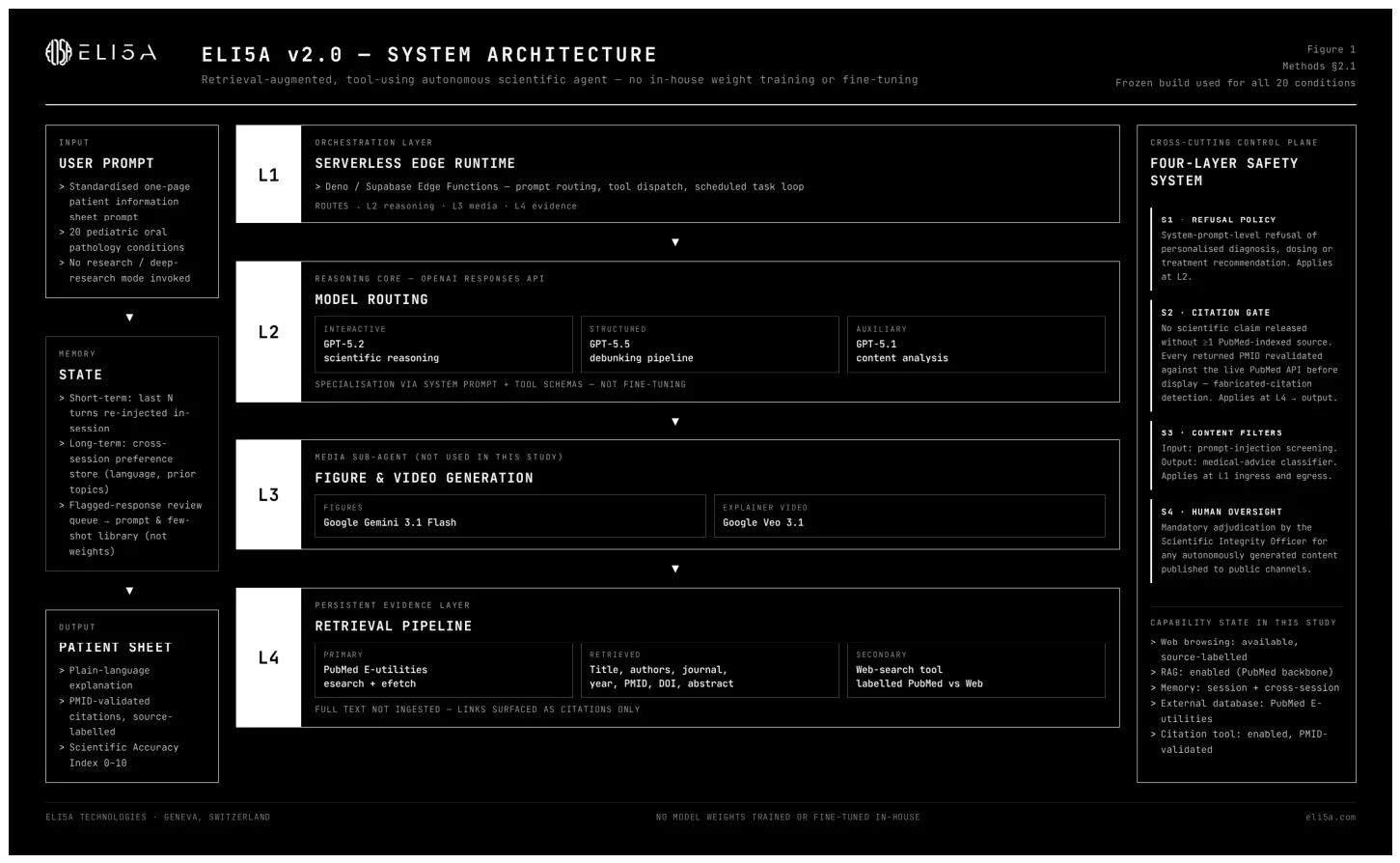
Architecture of the Eli5a v2.0 autonomous scientific agent. Figure 2 description. The system is a retrieval-augmented, tool-using agent; no model weights were trained or fine-tuned in-house. L1, orchestration layer on a serverless edge runtime; L2, reasoning core routing prompts across the OpenAI Responses API model family; L3, media sub-agent (not invoked in the present study, in which all outputs were text); L4, persistent evidence layer grounding every scientific claim in PubMed-indexed literature retrieved at inference time. The right-hand plane shows the four safety layers (S1–S4), which act across rather than within the pipeline, and the capability state of the agent during data collection. The left-hand column shows the standardized study prompt, the memory subsystem, and the output artefact evaluated by the three blinded raters.

Figure 1 therefore presents the complete proposed methodology and the flow of implementation across all evaluated techniques, rather than the internal design of any single platform. The architecture of the Eli5a v2.0 agent, which constitutes one component of Step 3 only, is presented separately in Figure 2, and the two figures are intended to be read together.

### 2.2. Development of the Autonomous AI Scientific Agent

To address limitations of conventional chatbot-style LLMs, a novel autonomous AI agent, Eli5a, was developed. Its name derives from “Explain Like I’m Five” (ELI5), an established convention for lay-level explanation of technical material [15]. Eli5a extends the phrase to “Explain Like I’m Five, Academia,” a designation introduced by the developers for lay explanations anchored to peer-reviewed literature [16]. The agent was developed to engage the public with scientific research through concise summaries and integration with social-media platforms. Its prompt library, retrieval pipeline, and few-shot exemplars were curated by a multidisciplinary scientific team using health-science literature indexed in PubMed.

Eli5a v2.0 is a retrieval-augmented, tool-using agent rather than a fine-tuned base model [17]. Its architecture comprises (i) an orchestration layer on a serverless edge runtime (Deno/Supabase Edge Functions); (ii) a reasoning layer that routes prompts through the OpenAI Responses API; (iii) a media sub-agent for scientific figures and short explanatory video, which was not used in this text-only study; and (iv) an evidence layer connected to PubMed E-utilities. The complete architecture and its cross-cutting safety controls are summarized in Figure 2. Because Eli5a is continuously deployed, “v2.0” refers to a configuration bundle comprising the system prompt, tool schemas, retrieval pipeline, and model-routing table rather than to a model checkpoint; no model weights were trained or fine-tuned in-house. All patient information sheets were generated using one frozen v2.0 configuration during the study data-collection window, without changes to the prompt library, few-shot exemplars, retrieval pipeline, or model routing. The system prompt instructs the agent to use language suitable for a general audience, decline personalized medical advice, cite peer-reviewed sources using PubMed identifiers, and display a platform-generated “Scientific Accuracy Index.” This index was not validated or evaluated as an outcome in the present study.

Eli5a v2.0 maintains short-term conversational memory within a session and preference memory across sessions. User-flagged responses are logged for review, and corrections are incorporated into the prompt and exemplar library rather than into model weights. For automated public content, the agent can retrieve new PubMed abstracts, generate plain-language summaries with citations, and post them after human review. It can also invoke web search for time-sensitive information, while PubMed remains its primary evidence source. At inference, the agent queries PubMed E-utilities and retrieves structured metadata and abstracts; full-text articles are not ingested, although available links may be displayed. The developers describe four safety layers: a refusal policy for personalized diagnosis or treatment; a citation requirement with live PMID validation; input and output content filters; and mandatory human oversight for autonomously generated public content. These architectural safeguards were described but were not independently validated as outcomes in this study.

### 2.3. Study Design

Patient education sheets were generated using five platforms: Eli5a v2.0 (Eli5a Technologies, Geneva, Switzerland; eli5a.com), ChatGPT-5.4 (OpenAI, San Francisco, CA, USA), Claude Sonnet 4.6 (Anthropic, San Francisco, CA, USA), Perplexity (Perplexity AI, Inc., San Francisco, CA, USA), and Doximity (Doximity, Inc., San Francisco, CA, USA). For each chatbot, the following prompt was applied uniformly across 20 pediatric oral pathology conditions: “Generate a one-page patient information sheet on each of the following topics:“ followed by the topics’ titles, without any additional feature (e.g., research mode).

Doximity is a professional medical network restricted to verified clinicians, and its generative writing assistant is offered within that credential-gated environment rather than as a consumer product; it is positioned for professional documentation and patient-communication tasks under clinician oversight. It was included here precisely because it is the only evaluated platform whose intended user is the clinician rather than the layperson. This provides an informative contrast: material generated in a clinician-facing environment might reasonably be expected to perform well on instruments assessing reliability and sourcing, while being pitched at a reading level above that recommended for patient education.

All evaluated platforms were tested under default, out-of-the-box configurations, with no manual adjustment of decoding parameters (temperature, top-p, or max-token limits); where such settings existed, they were vendor-controlled and not user-configurable. System prompts were similarly left unmodified: Eli5a v2.0 retained its built-in “ELI5A persona” prompt, while ChatGPT-5.4, Claude Sonnet 4.6, Perplexity, and Doximity ran with default settings and no custom instructions, prompts, or style customization applied. This ensured a standardized, un-customized basis for cross-platform comparison. Supplemental Table S1 illustrates the enabled features by AI chatbot platforms evaluated.

The generated patient information sheets were evaluated using four complementary instruments. The Global Quality Score (GQS) is a 5-point Likert scale used to assess the overall quality, flow, completeness, and usefulness of health information [18]. DISCERN is a 16-item, 5-point instrument covering publication reliability, information about treatment choices, and overall quality [19]. Understandability and actionability were evaluated using the Patient Education Materials Assessment Tool (PEMAT), which yields separate scores for how readily users can understand a resource and identify actions they can take [20]. The Flesch-Kincaid Grade Level was calculated as an objective readability estimate based on sentence length and word complexity, expressed as a U.S. school grade level [21].

Section 2 of DISCERN (items 9–15) evaluates information on treatment choices. For benign, developmental, or self-limiting conditions, the clinically legitimate management options were defined before scoring to include active surveillance, reassurance without intervention, and symptomatic or supportive care. A sheet was credited when it described these approaches, their benefits or risks, the natural history without intervention, and effects on quality of life. No item was treated as not applicable or omitted; all platforms were scored using the same 16-item instrument and constant maximum score. The three evaluators were briefed on this convention before scoring.

The 20 conditions were selected purposively to span pediatric oral pathologic diagnoses for which a parent may seek written information. Selection was led by a pediatric dental specialist (RR) and reviewed by two oral and maxillofacial pathologists (ZHK and ASS). For this study, common conditions were defined as diagnoses routinely encountered in pediatric dental or primary-care practice. Rare conditions were defined using established United States and European prevalence thresholds: fewer than 200,000 affected individuals in the United States or fewer than 1 in 2000 people in the European Union [22]. The complete list of conditions and the authoritative patient-information sources used as reference material are provided in Supplementary Material S1. Three evaluators (RR, KSK, and NN), none affiliated with the five evaluated platforms, assessed the generated sheets in a blinded manner using the prespecified instruments. Because the study involved no direct interaction with human participants and analyzed publicly available information, ethical approval was not required.

### 2.4. Statistical Analysis

Descriptive statistics, including the mean, median, standard deviation, minimum, and maximum, were calculated for each outcome and platform. For GQS, the three evaluators’ ratings were averaged for each condition, platform combination before platform comparisons. Because the same 20 conditions were evaluated across all five platforms, the observations were paired. Differences among platforms were assessed separately for GQS, DISCERN, PEMAT understandability, PEMAT actionability, and Flesch–Kincaid Grade Level using Friedman tests. Significant omnibus findings were followed by two-sided paired Wilcoxon signed-rank tests with Holm correction for the 10 pairwise comparisons. Kendall’s W was reported as the omnibus effect size. Interrater reliability for GQS was assessed using a two-way random-effects, absolute-agreement intraclass correlation coefficient (ICC), with ICC(2,1) representing single-evaluator reliability and ICC(2,3) representing the reliability of the mean of three evaluators. The 95% confidence intervals were estimated using 20,000 target-level bootstrap samples. Statistical significance was set at an adjusted *p* < 0.05. Analyses were conducted using Python (version 3.13.0) and SciPy (version 1.16.2).

## 3. Results

### 3.1. Global Quality Score

**Figure 3:**
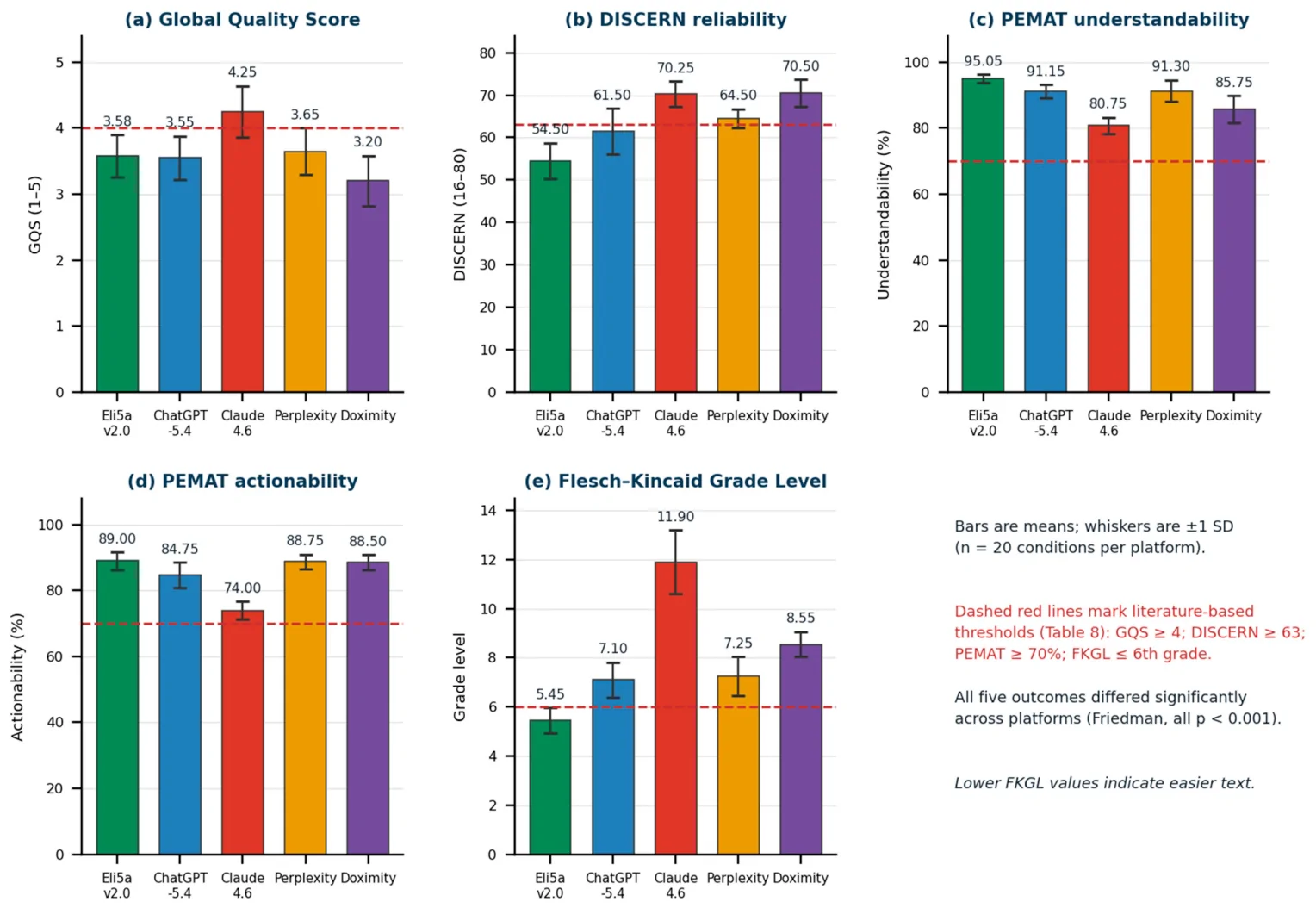
Comparative performance of the five evaluated platforms across the five outcome measures. Figure 3 description. Panels (**a**–**e**) show the Global Quality Score, the DISCERN score, PEMAT understandability, PEMAT actionability, and the Flesch–Kincaid Grade Level, respectively. Bars represent means and whiskers represent ±1 standard deviation across the 20 conditions evaluated on each platform. Dashed red lines mark the literature-based thresholds applied in Table 8: a GQS ≥ 4, a DISCERN score ≥ 63, PEMAT scores ≥ 70% for both subscales, and a Flesch–Kincaid Grade Level ≤ 6. In panel (**e**), lower values denote more accessible text, so a bar below the dashed line indicates compliance with the recommended reading level. All five outcomes differed significantly across platforms on Friedman testing (all *p* < 0.001).

GQS differed significantly across the five platforms (Friedman χ^2^(4) = 45.44, *p* < 0.001, Kendall’s W = 0.568). Claude 4.6 achieved the highest mean GQS (4.25 ± 0.39), followed by Perplexity (3.65 ± 0.35), Eli5a v2.0 (3.58 ± 0.32), ChatGPT-5.4 (3.55 ± 0.33), and Doximity (3.20 ± 0.38). Holm-adjusted paired comparisons showed that Claude 4.6 scored significantly higher than all other platforms (all adjusted *p* ≤ 0.002). Eli5a v2.0, ChatGPT-5.4, and Perplexity did not differ significantly from one another, while each scored significantly higher than Doximity (adjusted *p* = 0.009, 0.042, and 0.016, respectively), Table 1 and Figure 3a.

### 3.2. DISCERN Score

DISCERN scores differed significantly across platforms (Friedman χ^2^(4) = 62.31, *p* < 0.001, Kendall’s W = 0.779). Doximity (70.50 ± 3.20) and Claude 4.6 (70.25 ± 3.02) achieved the highest mean scores, followed by Perplexity (64.50 ± 2.24), ChatGPT-5.4 (61.50 ± 5.40), and Eli5a v2.0 (54.50 ± 4.26). Claude 4.6 and Doximity did not differ significantly (adjusted *p* = 0.796), and ChatGPT-5.4 and Perplexity did not differ after Holm correction (adjusted *p* = 0.064). All other pairwise comparisons were significant (adjusted *p* ≤ 0.005) (Table 2; Figure 3b).

### 3.3. PEMAT Score

PEMAT understandability differed significantly across platforms (Friedman χ^2^(4) = 65.67, *p* < 0.001, Kendall’s W = 0.821). Eli5a v2.0 achieved the highest mean score (95.05 ± 1.23), followed by Perplexity (91.30 ± 3.29), ChatGPT-5.4 (91.15 ± 2.06), Doximity (85.75 ± 4.06), and Claude 4.6 (80.75 ± 2.45). ChatGPT-5.4 and Perplexity did not differ significantly (adjusted *p* = 0.971); all other pairwise comparisons were significant (adjusted *p* ≤ 0.003) (Table 3; Figure 3c). PEMAT actionability differed significantly across platforms (Friedman ^2^(4) = 60.66, *p* < 0.001, Kendall’s W = 0.758). Eli5a v2.0 (89.00 ± 2.62), Perplexity (88.75 ± 2.22), and Doximity (88.50 ± 2.35) achieved the highest mean scores, followed by ChatGPT-5.4 (84.75 ± 3.80) and Claude 4.6 (74.00 ± 2.62). Eli5a v2.0, Perplexity, and Doximity did not differ significantly from one another. Each scored significantly higher than ChatGPT-5.4 and Claude 4.6, and ChatGPT-5.4 scored significantly higher than Claude 4.6 (all significant adjusted *p* ≤ 0.013) (Table 4; Figure 3d).

### 3.4. Flesch–Kincaid Grade Level

FKGL differed significantly across platforms (Friedman χ^2^(4) = 75.00, *p* < 0.001, Kendall’s W = 0.938). Eli5a v2.0 produced the lowest grade level (5.45 ± 0.51), followed by ChatGPT-5.4 (7.10 ± 0.72), Perplexity (7.25 ± 0.79), Doximity (8.55 ± 0.51), and Claude 4.6 (11.90 ± 1.29). ChatGPT-5.4 and Perplexity did not differ significantly (adjusted *p* = 0.083); all other comparisons were significant (adjusted *p* ≤ 0.001) (Table 5; Figure 3e).

Figure 3 presents these findings graphically. For each of the five outcomes, the group mean and the corresponding standard deviation are displayed alongside the literature-based threshold for that instrument, so that both the relative ranking of the platforms and their position with respect to accepted benchmarks can be appreciated visually.

### 3.5. Primary Omnibus Analyses

The primary omnibus analyses demonstrated statistically significant differences among the five AI platforms across all evaluated outcomes (Table 6). Friedman tests were significant for GQS, DISCERN, PEMAT understandability, PEMAT actionability, and FKGL, with all *p*-values < 0.001. Effect sizes varied from moderate to very large by Kendall’s W, indicating that platform identity accounted for a substantial proportion of the rank-order differences across outcomes. The largest omnibus effect was observed for FKGL (χ^2^ = 75.00, W = 0.938), followed by PEMAT understandability (χ^2^ = 65.67, W = 0.821), DISCERN χ^2^ = 62.31, W = 0.779), PEMAT actionability^2^(χ^2^ = 60.66, W = 0.758), and GQ^2^ (χ^2^ = 45.44, W = 0.568). These findings confirm that the evaluated platforms differed meaningfully not only in overall quality and reliability, but also in patient-centered communication domains and readability (Table 6).

**Figure 4:**
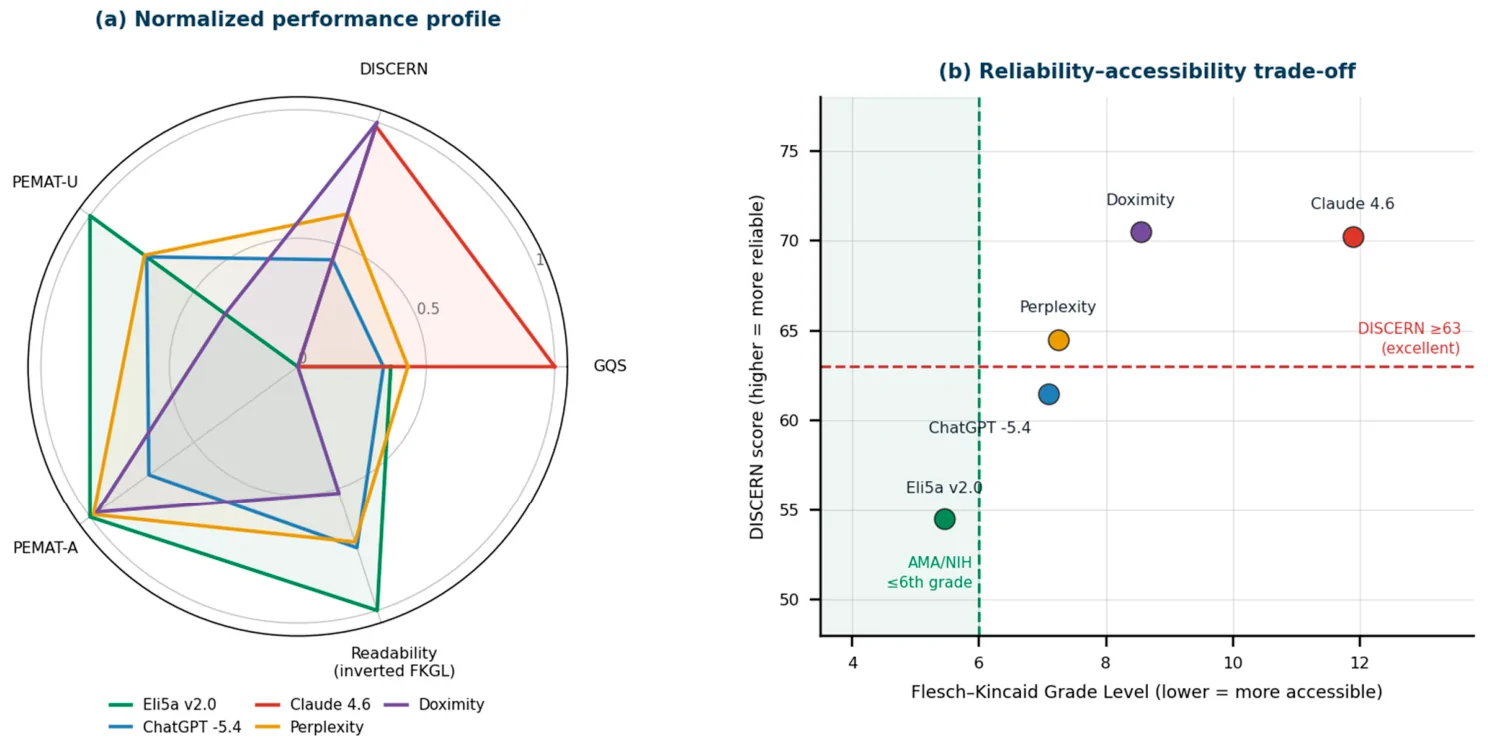
Multidomain performance profiles and the reliability–accessibility trade-off. Figure 4 description. Panel (**a**) shows the five outcome domains for each platform after min–max normalization across the evaluated platforms, with the Flesch–Kincaid Grade Level inverted so that a larger radius denotes better performance in every domain; the normalization is relative to the five platforms evaluated here and not to an absolute scale. Panel (**b**) plots the mean DISCERN score against the mean Flesch–Kincaid Grade Level for each platform. The vertical dashed line marks the AMA/NIH recommended ceiling of a sixth-grade reading level, with the shaded region to its left indicating compliance, and the horizontal dashed line marks the DISCERN threshold of 63 for excellent reliability. No platform occupied the upper-left region of the plot, which illustrates the trade-off between information reliability and lay accessibility described in the Discussion.

The multidomain pattern underlying these omnibus results is shown in Figure 4. The normalized profiles indicate that each platform occupied a distinct region of the outcome space, and the joint plot of reliability against readability shows that the platforms meeting the DISCERN threshold for excellence were separate from the single platform meeting the recommended sixth-grade reading level.

### 3.6. Interrater Reliability

Absolute agreement among the three evaluators for GQS was poor for a single evaluator (ICC(2,1) = 0.236, 95% bootstrap CI 0.100–0.367) and poor to moderate for the mean of three evaluators (ICC(2,3) = 0.481, 95% bootstrap CI 0.250–0.635). The previously reported Fleiss κ value of 0.91 was removed. Because interrater absolute agreement was lower than initially reported, GQS results based on average evaluator ratings should be interpreted with appropriate caution (Table 7).

**Table 7:** Interrater reliability for GQS. Absolute-agreement, two-way random-effects ICC across 100 targets (20 conditions × 5 platforms) rated by all three evaluators. Confidence intervals are percentile bootstrap intervals based on 20,000 target-level bootstrap samples.

Reliability Estimate	ICC	95% Bootstrap CI	Interpretation
Single evaluator: ICC(2,1)	0.236	0.100-0.367	Poor
Mean of three evaluators: ICC(2,3)	0.481	0.250-0.635	Poor to moderate

## 4. Discussion

Recent evidence suggests that frontier LLMs may support patient education by generating accessible medical information and explaining complex healthcare concepts [23]. In this study, Claude Sonnet 4.6 achieved the highest GQS and, together with Doximity, the highest DISCERN scores, but it ranked lowest in understandability, actionability, and readability. Conversely, Eli5a v2.0 achieved the highest understandability and the lowest and most ideal reading grade level; its actionability did not differ significantly from Perplexity or Doximity, and it had the lowest DISCERN score. These results show that the platforms had distinct strengths and weaknesses and that no single platform was superior across all evaluated domains. Importantly, the four instruments used here assess information quality, reliability, understandability, actionability, and readability; none directly assesses factual accuracy. Because no structured factual-accuracy or citation-verification audit was performed, the present data cannot support a claim that any platform’s output was free of factual errors or hallucinated content.

The study hypothesis was only partially supported. Eli5a v2.0 performed best on understandability and readability, but not on overall quality or reliability, and its actionability was statistically comparable with Perplexity and Doximity. Hallucination and citation fabrication are documented risks in medical applications of generative AI [24,25], but whether Eli5a’s retrieval and citation safeguards reduce such errors was not tested and remains a question for a dedicated accuracy audit. Previous patient-education studies have likewise shown that platform performance depends on both the task and the outcome being measured [26,27]. The present work extends an earlier evaluation of consumer LLMs in oral oncology by our group in three respects [28]. First, it evaluates complete patient information sheets rather than answers to individual questions. Second, it focuses on pediatric oral conditions and includes rare head and neck tumors for which lay-language resources are limited. Third, it directly compares a retrieval-grounded autonomous agent with frontier general-purpose platforms. The earlier study found that the highest-quality chatbot generated the least readable text, whereas the easiest-to-read tool provided less robust information. The same inversion was observed here: Claude Sonnet 4.6 led on quality and reliability but generated text at the highest grade level, whereas Eli5a v2.0 led on understandability and readability but had the lowest DISCERN score. Reproduction of this pattern across different condition sets, output formats, and platform generations supports interpreting it as a recurring quality-accessibility trade-off rather than as overall superiority of either system type.

When benchmarked against established thresholds rather than relative rank alone, a more nuanced picture emerges (Table 8). Claude 4.6 was the only chatbot to reach the GQS “high quality” threshold (≥4), and, along with Doximity and Perplexity, the DISCERN “excellent” threshold (≥63). However, all five chatbots exceeded the PEMAT acceptability threshold (≥70%) for both understandability and actionability, indicating that despite statistically significant differences among tools, none produced patient education material that would be considered unusable by this standard. In contrast, readability showed a clear pass/fail divide: only Eli5a v2.0 met the AMA/NIH-recommended ceiling of a 6th-grade reading level, while Claude 4.6—the top performer on quality and reliability—produced content nearly double the recommended grade level (11.90). The expectation that a clinician-facing platform would favor reliability over accessibility was borne out. Doximity achieved a DISCERN score statistically indistinguishable from that of Claude Sonnet 4.6 (70.50 ± 3.20 versus 70.25 ± 3.02) and met the DISCERN “excellent” threshold, yet recorded the lowest Global Quality Score of the five platforms (3.20 ± 0.38) and a mean reading level of 8.55 ± 0.51, above the recommended sixth-grade ceiling. Reliability scaffolding and lay accessibility are therefore separable properties, and a platform designed for clinician use should not be assumed to be suitable for direct distribution to families without adaptation. Certainly, this divergence underscores a meaningful trade-off: the highest-quality, most reliable content was also the least accessible to its intended pediatric-caregiver audience, raising the question of whether superior GQS and DISCERN performance translates into superior real-world patient comprehension when the material may exceed many caregivers’ reading level. This divergence is shown graphically in Figure 4b, in which no platform occupies the region combining excellent reliability with a reading level at or below sixth grade.

These findings are consistent with recent benchmark evidence that general-purpose frontier LLMs can outperform specialized clinical AI tools on broad medical tasks [29]. The present results add an important qualification: a purpose-built agent may show an advantage when its optimization target aligns with a communication-specific outcome. Here, the general-purpose model led on overall quality and reliability, whereas the communication-optimized agent led on understandability and readability. Domain specialization should therefore be evaluated against the specific property it was designed to improve rather than assumed to confer overall superiority. The omnibus results reinforce that AI-generated patient education materials cannot be evaluated using a single global performance metric. Significant platform-level differences were present for every outcome, with large to very large Kendall’s W values for DISCERN, PEMAT understandability, PEMAT actionability, and FKGL, suggesting consistent and clinically meaningful separation among platforms. The especially large effect for FKGL indicates that readability was the domain most strongly influenced by platform choice, while the moderate-to-large effect for GQS suggests greater overlap in perceived overall quality than in readability or patient-centered communication metrics. This pattern supports the central interpretation of the study: different platforms appear to optimize different aspects of patient information generation. Therefore, selection of an AI tool for pediatric oral pathology education should be guided by the intended use, such as reliability-focused clinician review versus first-contact caregiver-facing communication, rather than by an assumption that one platform is uniformly superior across all domains.

It must be stated that newer model versions for AI agents are now available with enhanced capabilities. The findings are anchored to the platform versions currently within a single data-collection window (21 April 2026–11 May 2026), and generative platforms are updated frequently. Absolute scores should therefore be regarded as version-specific rather than as stable properties of the products, and the rank orderings that were not statistically separable in the present analysis could plausibly reorder with subsequent releases. The larger and more consistent differences, notably readability and understandability, reflect deliberate differences in target audience and output style that vendors have maintained across releases, and are correspondingly less likely to invert. Periodic re-benchmarking is required if AI-generated patient education material is to be relied upon in practice, and the standardized prompt and instrument set used here are provided in the Supplementary Materials to make such re-benchmarking straightforward. Additionally, other LLMs designed for medical purposes (e.g., Med-PaLM, BioMedLM) that were not publicly available were not assessed. Moreover, the evaluated responses were generated in the English language, hence this study did not evaluate the potential for language-related disparities in healthcare information using underrepresented languages [30].

In summary, Eli5a v2.0’s advantage over the evaluated frontier platforms was specific rather than general. It was the only platform to generate material at or below the recommended sixth-grade reading level (5.45 ± 0.51) and achieved the highest understandability score (95.05 ± 1.23). Its actionability score was also high (89.00 ± 2.62) but was not significantly different from those of Perplexity or Doximity. In contrast, Eli5a v2.0 recorded the lowest DISCERN score (54.50 ± 4.26), and its GQS did not differ significantly from ChatGPT-5.4 or Perplexity. It is therefore not a better tool overall, but it may be better suited to first-contact explanations for lay caregivers. Future versions should improve visible reliability scaffolding by presenting retrieved sources and retrieval dates, explicitly covering legitimate management options including observation and supportive care, and directing families to further professional support. Prospective re-evaluation should determine whether these changes improve reliability without eroding readability and should include expert factual-accuracy review and direct caregiver-comprehension outcomes.

## 5. Conclusions

This study directly compared a purpose-built AI agent (Eli5a v2.0) with four frontier LLM platforms for generating pediatric oral pathology patient information sheets. No platform excelled across all domains: Claude Sonnet 4.6 led in overall quality and, together with Doximity, reliability, whereas Eli5a v2.0 produced the most understandable and readable material and was the only platform meeting the recommended reading-level standard. The central finding was a trade-off between information quality and reliability on one hand and lay accessibility on the other across five measures and 20 conditions. Communication-optimized agents may therefore offer an advantage for first-contact explanations, particularly for rare pediatric conditions for which accessible information is limited, but the present study did not demonstrate overall superiority or directly assess factual accuracy or caregiver comprehension. Platform choice should match the communication task. A two-stage workflow in which an accessible draft undergoes factual and clinical review is more defensible than reliance on any single platform, and all AI-generated materials require clinician review before distribution to families.

Limitations include poor-to-moderate interrater agreement on GQS, single-occasion evaluation by three raters without patient or parent input, no factual-accuracy or citation audit, unequal retrieval and browsing capabilities across platforms, English-only outputs tied to a single data-collection window, and author affiliations with one platform’s developer, although the blinded evaluators had no such affiliations. DISCERN was developed to appraise treatment-choice information and may be less discriminating for benign or self-limiting conditions that require no active treatment; despite a uniform scoring convention, some differences may reflect how explicitly platforms framed observation or supportive care. Conditions were purposively rather than randomly sampled and were pooled across common and rare strata, so whether platform rankings differ by condition rarity was not tested. Future priorities include configuration-matched comparisons, formal expert accuracy and citation audits against primary sources, caregiver-comprehension studies, non-English evaluation, and prospective re-benchmarking as platforms evolve. For Eli5a v2.0 specifically, the central development goal is to improve reliability without sacrificing readability.

## Figures and Tables

**Table 1:** Descriptive statistics for GQSs by chatbot (n = 20 per chatbot, scores averaged across 3 graders).

Chatbot	Mean	Median	SD	Min	Max
Eli5a v2.0	3.58	3.50	0.32	3.00	4.00
ChatGPT-5.4	3.55	3.67	0.33	3.00	4.00
Claude 4.6	4.25	4.33	0.39	3.67	5.00
Perplexity	3.65	3.67	0.35	3.00	4.33
Doximity	3.20	3.33	0.38	2.33	3.67

**Table 2:** Descriptive statistics for DISCERN scores by chatbot (n = 20).

Chatbot	Mean	Median	SD	Min	Max
Eli5a v2.0	54.50	55.00	4.26	50	65
ChatGPT-5.4	61.50	60.00	5.40	55	75
Claude 4.6	70.25	70.00	3.02	65	75
Perplexity	64.50	65.00	2.24	60	70
Doximity	70.50	70.00	3.20	60	75

**Table 3:** Descriptive statistics for Understandability (PEMAT-U) scores by chatbot (n = 20).

Chatbot	Mean	Median	SD	Min	Max
Eli5a v2.0	95.05	95.00	1.23	93	98
ChatGPT-5.4	91.15	90.00	2.06	90	95
Claude 4.6	80.75	80.00	2.45	75	85
Perplexity	91.30	90.00	3.29	85	97
Doximity	85.75	85.00	4.06	80	95

**Table 4:** Descriptive statistics for Actionability (PEMAT-A) scores by chatbot (n = 20).

Chatbot	Mean	Median	SD	Min	Max
Eli5a v2.0	89.00	90.00	2.62	85	95
ChatGPT-5.4	84.75	85.00	3.80	80	90
Claude 4.6	74.00	75.00	2.62	70	80
Perplexity	88.75	90.00	2.22	85	90
Doximity	88.50	90.00	2.35	85	90

**Table 5:** Descriptive statistics for Flesch–Kincaid Grade Level (FKGL) scores by chatbot (n = 20).

Chatbot	Mean	Median	SD	Min	Max
Eli5a v2.0	5.45	5.00	0.51	5	6
ChatGPT-5.4	7.10	7.00	0.72	6	8
Claude 4.6	11.90	13.00	1.29	10	13
Perplexity	7.25	7.00	0.79	6	8
Doximity	8.55	9.00	0.51	8	9

**Table 6:** Primary omnibus analyses.

Outcome	Friedman χ^2^	df	*p*-Value	Kendall’s W
GQS	45.44	4	<0.001	0.568
DISCERN	62.31	4	<0.001	0.779
PEMAT understandability	65.67	4	<0.001	0.821
PEMAT actionability	60.66	4	<0.001	0.758
FKGL	75.00	4	<0.001	0.938

**Table 8:** Literature-Based Performance Thresholds.

Metric	Literature Benchmark	Source	Chatbots Meeting Threshold	Chatbots Below Threshold
GQS	≥4 = high quality (1–2 poor, 3 moderate)	Bernard et al. [18] GQS scale	Claude 4.6 (4.25)	Perplexity (3.65), Eli5a v2.0 (3.58), ChatGPT-5.4 (3.55), Doximity (3.20)—all moderate
DISCERN	≥63 = excellent (51–62 good, 39–50 fair)	Charnock et al. [19], DISCERN handbook	Claude 4.6 (70.25), Doximity (70.50), Perplexity (64.50)	ChatGPT-5.4 (61.50), Eli5a v2.0 (54.50)—both good, not excellent
PEMAT-U	≥70% acceptable	Shoemaker et al. [20]	All five (80.75–95.05)	None
PEMAT-A	≥70% acceptable	Shoemaker et al. [20]	All five (74.00–89.00)	None
FKGL	≤6th grade	AMA/NIH readability guidelines	Eli5a v2.0 (5.45)	Claude 4.6 (11.90), Doximity (8.55), Perplexity (7.25), ChatGPT-5.4 (7.10)

## Data Availability

Data available on request from the authors.

## Supplementary Materials

The following supporting information can be downloaded at: https://www.mdpi.com/article/10.3390/cancers18183047/s1: Table S1, enabled features and capability disclosure for the evaluated AI platforms; Supplementary Material S1, evaluated conditions and authoritative reference sources.
