# Supplementary material for: Comparative Evaluation of a New Autonomous AI Agent Versus Frontier LLMs for AI-Generated Patient Information Sheets on Pediatric Pathologies
Source: Cancers (Basel). 2026 Sep 20;18(18):3047. doi: 10.3390/cancers18183047 (PMC13604920; doi:10.3390/cancers18183047)
Supplement: Supplementary file 1 [file cancers-18-03047-s001.zip › cancers-4532241-supplementary.pdf]

**Supplementary Material S1**

Pediatric oral pathology topics and their resources

1. Eruption cyst

1. https://my.clevelandclinic.org/health/diseases/eruption-cyst
2. https://www.colgate.com/en-us/oral-health/mouth-sores-and-infections/eruptioncyst-causes-symptoms-and-treatments

2. Natal and neonatal teeth

1. https://my.clevelandclinic.org/health/symptoms/natal-teeth
2. https://www.colgate.com/en-us/oral-health/kids-oral-care/importance-of-caringfor-natal-teeth

3. Congenital epulis

1. https://www.connecticutchildrens.org/specialties-conditions/fetal-carecenter/fetal-care-conditions/epignathus-epulis
2. https://rarediseases.org/mondo-disease/congenital-epulis/

4. Bohn's nodules

1. https://my.clevelandclinic.org/health/diseases/bohn-nodules
2. https://mdsearchlight.com/child-health/palatal-and-gingival-cysts-of-the-newbornepstein-pearls-bohn-nodules/

5. Epstein's pearls

1. https://my.clevelandclinic.org/health/diseases/epstein-pearls
2. https://www.webmd.com/parenting/baby/what-are-epstein-pearls

6. Dental lamina cysts

1. https://www.news-medical.net/health/Gingival-Cysts-in-Newborns.aspx

7. Mucocele

1. https://my.clevelandclinic.org/health/diseases/23406-oral-mucocele
2. https://www.colgate.com/en-us/oral-health/mouth-sores-andinfections/mucoceles-and-how-to-treat-them

8. Ranula

1. https://my.clevelandclinic.org/health/diseases/23451-ranula
2. https://www.chop.edu/conditions-diseases/ranula

9. Irritation fibroma

1. https://my.clevelandclinic.org/health/diseases/23991-fibroma
2. https://www.colgate.com/en-us/oral-health/mouth-sores-and-infections/fibromatreatment

10. Pyogenic granuloma

1. https://my.clevelandclinic.org/health/diseases/22717-pyogenic-granuloma
2. https://emedicine.medscape.com/article/1077040-overview

11. Peripheral ossifying fibroma

1. https://dermnetnz.org/topics/peripheral-ossifying-fibroma
2. https://emedicine.medscape.com/article/1077040-overview

12. Peripheral giant cell granuloma

1. https://emedicine.medscape.com/article/1077040-overview

13. Lymphangioma

1. https://my.clevelandclinic.org/health/diseases/23141-lymphangioma
2. https://www.childrens.com/specialties-services/conditions/lymphangioma

14. Hemangioma

1. https://mdsearchlight.com/oral-health/oral-hemangiomas/
2. https://my.clevelandclinic.org/health/diseases/23365-hemangioma

15. Ameloblastoma

1. https://www.mayoclinic.org/diseases-conditions/ameloblastoma/symptomscauses/syc-20351278
2. https://my.clevelandclinic.org/health/diseases/22143-ameloblastoma

16. Odontogenic keratocyst (OKC)

1. https://www.news-medical.net/health/Odontogenic-Keratocyst-(OKC).aspx

2. https://www.leedsth.nhs.uk/patients/resources/maxillofacial-surgery-treatment-ofodontogenic-keratocysts/

17. Dentigerous cyst

1. https://my.clevelandclinic.org/health/diseases/dentigerous-cyst

2. https://www.webmd.com/oral-health/what-are-dentigerous-cysts

18. Radicular cyst

1. https://www.news-medical.net/health/Radicular-Cysts.aspx

19. Hand, foot, and mouth disease

1. https://www.mayoclinic.org/diseases-conditions/hand-foot-and-mouthdisease/symptoms-causes/syc-20353035

2. https://www.cedars-sinai.org/health-library/diseases-and-conditions--pediatrics/h/hand-foot-and-mouth-disease-in-children.html

20. Melanotic neuroectodermal tumor of infancy (MNTI)

1. https://mdsearchlight.com/health/melanotic-neuroectodermal-tumor-of-infancy/

**<u>Supplemental Table S1.</u>** Enabled Features by AI Chatbot Platforms Evaluated. *Capability disclosure. Where a capability is recorded as "platform default, not disabled", this reflects the interface as used: no setting was altered from its out-of-the-box state.*

| **Platform** | **Web browsing** | **RAG** | **Memory** | **External database** | **Citation tool** |
|---|---|---|---|---|---|
| Eli5a v2.0 | Secondary tool; output sourcelabelled (PubMed vs. Web) | Enabled — primary architecture | Enabled — insession and cross-session | PubMed E-utilities; abstracts only, no full text | Enforced — ≥1 PubMed source per claim; every PMID revalidated live |
| ChatGPT-5.4 | Platform default, not disabled | Not userconfigurable | Platform default, not disabled | None declared | Only where the platform emits them |
| Claude Sonnet 4.6 | Platform default, not disabled | Not userconfigurable | Platform default, not disabled | None declared | Only where the platform emits them |
| Perplexity | Enabled by design — retrieval-first | Enabled by design | Platform default, not disabled | Open web index | Enabled by design — inline attribution |
| Doximity | Platform default, not disabled | Not userconfigurable | Platform default, not disabled | None declared | Only where the platform emits them |
